# Applying evidence-based medicine in general practice: a video-stimulated interview study on workplace-based observation

**DOI:** 10.1186/s12875-019-1073-x

**Published:** 2020-01-08

**Authors:** Lisanne S. Welink, Kaatje Van Roy, Roger A. M. J. Damoiseaux, Hilde A. Suijker, Peter Pype, Esther de Groot, Marie-Louise E. L. Bartelink

**Affiliations:** 1 Julius Center for Health Sciences and Primary Care, University Medical Center Utrecht, Utrecht University, Universiteitsweg 100, 3584 CX Utrecht, The Netherlands; 2 Department of Public Health and Primary Care, Ghent University, Corneel Heymanslaan 10, B-9000 Ghent, Belgium

**Keywords:** Evidence-based medicine, General practice, Family medicine, Workplace-based learning, Observational learning, Video-stimulated elicitation interviews

## Abstract

**Background:**

Evidence-based medicine (EBM) in general practice involves applying a complex combination of best-available evidence, the patient’s preferences and the general practitioner’s (GP) clinical expertise in decision-making. GPs and GP trainees learn how to apply EBM informally by observing each other’s consultations, as well as through more deliberative forms of workplace-based learning. This study aims to gain insight into workplace-based EBM learning by investigating the extent to which GP supervisors and trainees recognise each other’s EBM behaviour through observation, and by identifying aspects that influence their recognition.

**Methods:**

We conducted a qualitative multicentre study based on video-stimulated recall interviews (VSI) of paired GP supervisors and GP trainees affiliated with GP training institutes in Belgium and the Netherlands. The GP pairs (*n* = 22) were shown fragments of their own and their partner’s consultations and were asked to elucidate their own EBM considerations and the ones they recognised in their partner’s actions. The interview recordings were transcribed verbatim and analysed with NVivo. By comparing pairs who recognised each other’s considerations well with those who did not, we developed a model describing the aspects that influence the observer’s recognition of an actor’s EBM behaviour.

**Results:**

Overall, there was moderate similarity between an actor’s EBM behaviour and the observer’s recognition of it. Aspects that negatively influence recognition are often observer-related. Observers tend to be judgemental, give unsolicited comments on how they would act themselves and are more concerned with the trainee-supervisor relationship than objective observation. There was less recognition when actors used implicit reasoning, such as mindlines (internalised, collectively reinforced tacit guidelines). Pair-related aspects also played a role: previous discussion of a specific topic or EBM decision-making generally enhanced recognition. Consultation-specific aspects played only a marginal role.

**Conclusions:**

GP trainees and supervisors do not fully recognise EBM behaviour through observing each other’s consultations. To improve recognition of EBM behaviour and thus benefit from informal observational learning, observers need to be aware of automatic judgements that they make. Creating explicit learning moments in which EBM decision-making is discussed, can improve shared knowledge and can also be useful to unveil tacit knowledge derived from mindlines.

## Background

Applying evidence-based medicine (EBM) in practice – defined as combining clinical expertise, patient preferences and the best-available evidence when making decisions for individual patients – is important but hard to do [1–3]. EBM is taught according to the five steps defined in the Sicily Statement: ask, acquire, appraise, apply and evaluate [4]. General practice (GP) specialty training focuses on the first three steps: asking the right questions, searching for evidence and appraising that evidence [4–6]. However, to provide best care for individual patients, EBM training should also focus on EBM behaviour: learning to judiciously weigh the best available evidence in combination with the patient’s preferences, and one’s own clinical expertise, leading to an individual decision that is well-grounded [3, 4, 7–10]. Currently, the best way to learn EBM behaviour in the workplace is unknown. One study in GP specialty training showed that an intervention involving clinically integrated EBM training for trainees and supervisors did not lead to improved EBM behaviour among trainees in the workplace [11]. To optimise workplace-based EBM learning, we need greater insight into the learning processes in the workplace.

GP supervisors and trainees learn informally from each other while working together in the practice [12]. Presumably, EBM behaviour is also learned this way. Observation is a part of informal learning as the observer, either the supervisor or trainee, learns from viewing the other person execute a certain skill or task [12]. Medical education and cognitive psychology literature theorises that observation leads to learning by stimulating reflective and prospective deliberation: the observer reflects on the effectiveness of various strategies and thinks about this in light of their own goals and future actions [13–16]. Observational, opportunistic learning can be seen as complementary to deliberative learning strategies in the workplace, such as discussing a topic or a skill [12, 17, 18]. One study suggests that informal learning may be even more powerful than formal learning since it leads to socialisation and tacit knowledge, which can overrule explicit knowledge [19]. Delving deeper into the role of observational EBM learning is an essential component of acquiring insight into current learning processes in the workplace.

However, during consultations with patients, GPs and GP trainees alike take many decisions without making all their considerations explicit, which can make EBM behaviour hard to observe [20]. Recognising the argument behind a certain decision is important to enable the observer to reflect and thus actually learn from the observation. When the correct ‘why’ of the decision cannot be constructed or recognised, the observer might infer erroneous personal constructs or knowledge, which could lead to an incorrect application of the observed EBM behaviour in the future [19, 21]. However, the actual quality of such a decision is subordinate at that point: as long as an observer is able to recognise the actor’s use of the three EBM-elements, reflection is possible and learning can take place.

This study aimed to obtain deeper insight into observational learning of EBM behaviour. We investigated the extent to which GPs and GP trainees recognise each other’s EBM behaviour through observation, and identified the aspects that influence recognition. It is explicitly not our aim to judge good or poor EBM behaviour, but to investigate whether observers were able to recognise argumentation for decision-making, leading to learning possibilities. Our findings may provide a greater understanding of how observational learning of EBM behaviour takes place in the workplace.

## Method

### Study setting

This study was conducted in several general practices in the Netherlands and in Flanders, Belgium. In each practice, a GP trainee works alongside a GP supervisor, both of whom participated in this study as a pair. GP specialty training in the Netherlands and in Flanders is comparable postgraduate medical training. However, most trainees in the Netherlands gain some working experience before starting GP specialty training, whereas most Belgian trainees start postgraduate training following their undergraduate track.

In both countries, training includes two years of working alongside a GP: Dutch trainees stay one year at most in the same practice. Belgian trainees can choose to work with the same GP for two years. Formal education in both countries is done at training institutes in small-group classes; EBM training is a common topic in these classes. Supervisors receive formal training (including EBM) in teach-the-teacher sessions.

### Study design and participant recruitment

A qualitative multicentre study was conducted using video-stimulated elicitation interviews (VSI) of pairs of GPs and GP trainees affiliated with GP training institutes in Antwerp or Ghent, Belgium or Utrecht, the Netherlands. Potential participants were approached between September 2016 and April 2017. We presented information about the study on a website, handed out flyers and gave promotional speeches at the training institutes. In Flanders, we could use purposeful sampling to maximise variation [22]. Recruitment in the Netherlands was harder, which meant we had to switch to convenience sampling there. Following recruitment, participants filled out a short questionnaire on baseline characteristics.

### Data collection

Data collection took place between November 2016 and August 2017. We recorded an average of ten random daily practice consultations per participant. One author (LW) selected two suitable consultation fragments per participant to be shown at the VSI. Fragments were considered suitable when decision-making of the participant was observed. The medical content of a consultation was never a selection criterion. To enhance recall, the semi-structured VSI were scheduled to take place within two weeks of the recordings [23–26]. The interviews followed a guide developed and iteratively revised by the research team (Additional file 1). Interviews were held individually and consecutively to ensure that the members of the pair could not influence each other. The interview consisted of two parts. In the first, the participant (either a supervisor or trainee) was shown two fragments of their own consultations and was asked to recall all their considerations for the decision(s) observed. They were asked to reveal the role that each of the three pillars of EBM (best evidence, patient values, and clinical expertise) had played in their decision-making and to mention any other factors that may have influenced the decision. In the second part, the participant was shown two fragments of decision-making by the other member of the pair (supervisor or trainee). They were asked to explicate the EBM considerations they recognised their supervisor or trainee making. Recognition of argumentation within all three pillars of EBM was questioned and extensively discussed. In the follow-up interview the other member was shown the same fragments to enable comparative within-case analysis. Interviews lasted approximately 45 to 60 min and took place in private at the GP’s surgery.

### Analysis

**Fig. 1:**
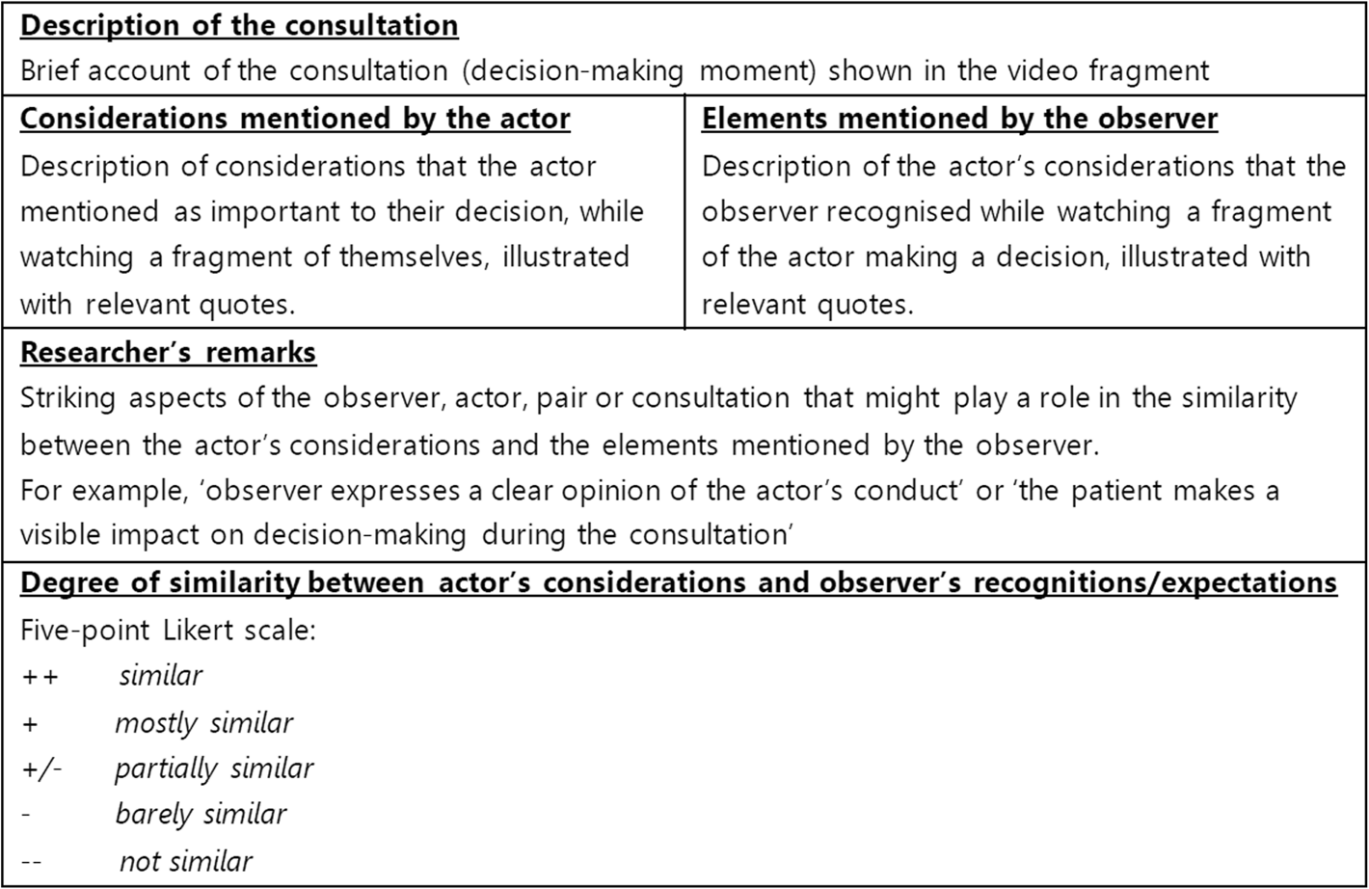
Structured template for analysis

All interviews were audio-recorded. The audio recordings were transcribed verbatim. To facilitate analysis, a template was developed (Fig. 1) to structure the findings. Each filled-in template contained background information and important remarks on the fragment and listed the considerations the acting supervisor/trainee (‘actor’) expressed during the interview as well as the elements the observing supervisor/trainee (‘observer’) mentioned while observing the same fragment. Put together, the video fragment information, the comments by both actor and observer, and the researcher’s remarks were considered one ‘case’, which enabled within-case comparison. Templates for each fragment were filled in separately by pairs of researchers. To enhance reflexivity, the composition of the researcher pairs rotated (LW, KVR, HS, EdG and MLB). All individual coding was extensively discussed within these pairs until consensus was reached. In the last step, the researcher pairs judged the degree of similarity between the actor and observer’s arguments according to a five-point Likert scale. Four templates were filled in per GP pair (two for the trainee as actor and two for the supervisor as actor).

Next, comparative case analysis was performed using NVivo 11 Pro software. To create a model describing aspects that influence the recognition of the actor’s EBM behaviour by the observer, we selected outlying pairs, i.e. those in which the actor’s and observer’s considerations were clearly similar or different. A pair was defined as ‘high in similarity’ (HS) when at least three out of four of their cases were labelled completely similar (++) or mostly similar (+). Inversely, a pair was defined as ‘low in similarity’ (LS) when at least three out of four of their cases were labelled as barely similar (−) or not similar (− −). In the final step of analysis, we identified aspects related to observer, actor, pair or consultation that were strikingly different between the two groups (HS and LS pairs). We decided to set the cut-off point at aspects coded at least 20% more often for one group than the other, because this difference seemed to be practically relevant [27].

### Ethical considerations

Approval was granted by the Ethical Board of the NVMO (Dutch Society of Medical Education) under case number 706. All GPs and GP trainees gave written informed consent to record their consultations and the interviews. At each consultation the GP supervisor or trainee asked the patient for their permission to be audio-recorded; during the video-recording only the physician was visible. The videos were uploaded via a secure connection to a secure electronic environment. Transcripts were anonymised and each pair was given a code number that still enabled participants to be identified as Dutch or Flemish and as trainee or supervisor.

## Results

The participants were thirteen Flemish and nine Dutch pairs who differed in supervising experience, experience in general practice, training stage and practice type (Table 1). The Flemish and Dutch pairs were comparable on these characteristics except on age.

**Table 1:** Characteristics of participants

	The Netherlands		Belgium	
	GP supervisors *n* = 9	GP trainees *n* = 9	GP supervisors *n* = 13	GP trainees *n* = 13
Female	3 ^a^	6	8	11
Age (average in years (range))	56 (48–67)	30 (28–35)	47 (36–57)	26 (25–30)
PhD trajectory (finished or ongoing)	1	2	1	0
Trainee in first year of training	5		8	
Trainee in last year of training	4		5	
GP supervisor’s experience as GP (average in years (range))	26 (20–38)		21 (12–33)	
GP’s experience as supervisor (average in years (range))	11 (2–20)		10 (2–25)	
Duration of collaboration between supervisor and trainee (average in months (range))	6 (4–9)		9 (3–18)	
Practice type				
Solo	0		2	
Duo	7		2	
Health centre	2		9	
Location of training institute				
Utrecht	9			
Antwerp	3			
Ghent	10			

^a^*Results are numbers, unless stated otherwise*

In total 44 individual interviews were held with 22 supervisor-trainee pairs. Within-case analyses were done on 85 cases in total, since four video fragments per pair were usually discussed in the interviews. Three pairs discussed only three video fragments during the interviews due to lack of time. Within-case analysis showed moderate similarity overall between the actor’s EBM behaviour and the observer’s recognition of this behaviour (Table 2), showing the same distribution on degree of similarity between Dutch and Belgian cases.

**Table 2:** Final judgement on degree of similarity, based on consensus by at least two researchers

Degree of similarity	Total number of cases (*n* = 85)	Dutch cases (*n* = 34)	Belgian cases (*n* = 51)
Similar (++)	4	1	3
Mostly similar (+)	13	5	8
Partially similar (+/−)	36	14	22
Barely similar (−)	26	12	14
Not similar ( - - )	6	2	4

**Fig. 2:**
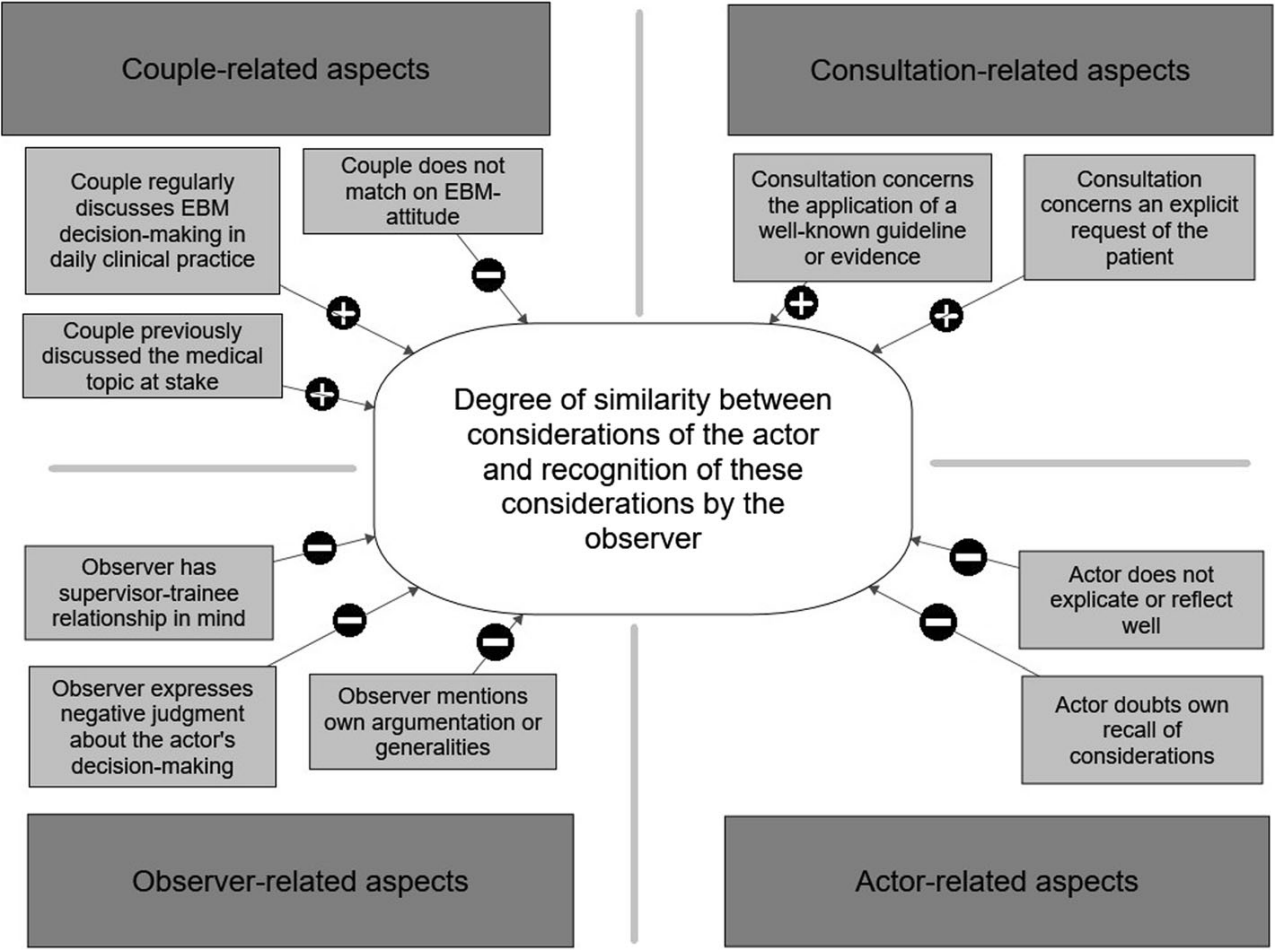
Aspects influencing similarity between the actor’s EBM behaviour and recognitions of the observer when observing consultations. + =  positively influencing degree of similarity - =  negatively influencing degree of similarity

Figure 2 presents a model describing the aspects influencing similarity between the actor’s EBM behaviour and the observer’s recognition of this behaviour. The four main aspects are divided in major themes that positively or negatively influence the degree of similarity.

### Pair-related aspects

In pairs low on similarity, the supervisor and trainee often had different attitudes to EBM. This was most apparent when a trainee felt it was important to follow the latest evidence or guidelines, whereas the supervisor preferred to rely on their experience. As a result, EBM-minded trainees could not recognise the experience-based considerations of their supervisors, and vice versa, supervisors relying on experience had difficulty recognising their trainee’s considerations that were based on the latest evidence.

**Table:** 

***Trainee****: [...] The supervisor and older doctors, they have really lots of experiential knowledge which is definitely good, but I think […] we graduated with lots of confidence in evidence-based medicine. It’s been pumped into us that it’s really important. So I’d rather follow the guideline than [...].* (Pair 16. All cases labelled ‘barely similar’, supervisor with > 30 years of experience as GP)	

When the supervisor and trainee had previously discussed the medical topic related to the observed consultation, the observer generally recognised the actor’s considerations better. Consistent with this finding, our analysis showed that when the working environment in the GP’s surgery is focused on regular discussions of EBM decisions, observers recognised the actor’s considerations more often.

**Table:** 

***Observing supervisor****: Again, I think it’s because we’ve already […]. So yes, children with fever is something you’d naturally discuss with the trainee. We’ve also gone through the guideline together. [...] I think she does it on that basis.* (Supervisor pair 21. Case labelled ‘mostly similar’)	

### Consultation-related aspects

The medical content of a decision made in a consultation seems to play only a marginal role in the recognition of another person’s EBM behaviour. A wide range of medical cases was shown during the interviews and no connection could be found between specific medical topics and the degree of recognition of the other person’s considerations about these topics.

The only content-related factor that seems to enhance recognition of EBM behaviour in consultations is when someone applies a well-established guideline or evidence. Pairs who individually or collaboratively obtained the same background information, such as knowledge from the common guideline on pain management, were able to recognise the other person’s use of this knowledge, even when it was applied implicitly. Previously discussed ‘common practices’, such as referrals to a certain hospital, were also easily recognised. Probably shared background knowledge makes recognition of considerations easier.

**Table:** 

***Acting trainee:*** *I got it from the guideline, that many children respond to viral infections with wheezing. That the airways contract a bit and then the treatment for that is a puffer, especially Ventolin. That’s what the guideline says, [you should prescribe Ventolin] from once up to four times a day.* ***Observing supervisor:*** *She decides on the basis of clinical research when to regard wheezing as the first symptom and then follows the guideline to prescribe Ventolin.* (Pair 19. Case labelled ‘completely similar’)	

A patient asking for more information during the consultation leads to better recognition by the observer. Probably the explicit request forces the actor to explain (aspects of) their considerations, which not only improves shared decision-making but also leads to more correct interpretations of EBM behaviour.

**Table:** 

*[Conversation between trainee and patient’s father during consultation]* **Acting trainee (to patient):** *“So yes, if we’re going to follow the guideline, I’d give you antibiotics again.”* **Patient’s father:** *“No. No, that doesn’t seem right. In my opinion we can still suppress it with paracetamol.”* **Observing supervisor:** “*So, with some reservation, she advises antibiotics but then the father says, let’s wait a bit longer [....] So yes, that leads to us doing what the father wants.”* (Pair 5. Case labelled ‘partially similar’)	

### Observer-related aspects

Our analysis showed that observer-related aspects influence recognition of EBM behaviour the most, no matter whether the trainee or the supervisor was observing. The act of observing and recognising the line of reasoning behind what is observed seems difficult for many observers. We can conclude this because observers not only appear to ‘observe’ different elements and reasoning from what the actors name, but often seem to engage in other activities than observation. Observers quickly became judgemental, gave unsolicited comments on how they would act in similar situations or spoke of how such decisions should be made in general. Others expressed confusion at having to explain someone else’s argument. Overall, in all cases with little or no similarity, the observer gave a negative opinion of the actor’s decision.

**Table:** 

**Observing trainee:** *I don’t know why he [the acting supervisor] said ‘week’. […] You’d expect to see some hyper-reactivity six weeks after a respiratory tract infection, and that man [the patient] confirmed that, of course. I don’t think I’d mention a time period. I’d say, well yes, I expect it [the symptoms] will ease and fade away eventually and I’d give some tips. But I don’t know why he [actor] said one week.* (Pair 3. Case labelled ‘partially similar’)	

Although both supervisors and trainees tended to express judgements and their own arguments instead of the considerations of the other physician, specific difficulties could be seen between trainee-observers and supervisor-observers. It seemed that supervisors often observe with their supervisor-trainee relationship in mind and appeared to see their main task as giving feedback on the trainee’s decisions and performance. Moreover, supervisors seem to interpret the trainee’s decision-making as driven by the trainee’s lack of knowledge or skills, even if the trainee sometimes appeared to have clear motives for their decision.

**Table:** 

**Observing supervisor:** *She also says, I find it too soon for an injection. I think it’s still something [...] she’s not up to doing an injection yet, not independently, not without involving me. That’s still a bit […] She can do it already but just, yes, under supervision. So I think that also plays a role.* **Acting trainee:** *I thought yes, he just needs a week of NSAIDs, and if that doesn’t work, then maybe keep him on NSAIDs a bit longer, and if that still doesn’t work, get him some support from the physio and then if that still doesn’t work well, then the injection. Those are the standard steps.* (Pair 2. Case labelled ‘not similar’)	

On the other hand, trainees seemed to find it hard to recognise their supervisor’s considerations if they thought their supervisor was not working according to the latest evidence. In this case trainees quickly formed a negative judgement on the actor’s decisions and felt obliged to elucidate their own reasoning.

**Table:** 

**Observing trainee:** *In this case I find it harder to understand the decisions he makes. [...] Switching to antibiotics after only three days without a fever, without an objective [check of] the infection parameters, actually I don’t find that… No, I wouldn’t do that.* **Interviewer:** *Why do you think he did it? What did he base [his decision] on?* **Trainee:** *No idea.* (Pair 8. Case labelled ‘not similar’)	

### Actor-related aspects

Another striking phenomenon revealed in the interviews was that when actors were watching their own fragments, they were often unable to repeat or reflect on their EBM behaviour during decision-making, even when explicitly asked to do so. Related to this, the actors also doubted their recollection of their own considerations and were unsure of their argumentation. The EBM behaviour of actors who had problems recalling their own substantiations was harder to distinguish and consequently there was less similarity between the actor’s considerations and the recognitions of the observer.

**Table:** 

**Interviewer:** *Why did you say, I recommend a nasal spray?* **Acting supervisor:** *Perhaps… ah yes, I don’t really know why. Perhaps because the side effect of that medicine is drowsiness and he gets rather tired during the day, perhaps that’s why. Ah yes, no idea. I no longer know why I said that.* **Observing trainee:** *So I think the decision [to prescribe] a nasal spray and those pills is based on experience. But it’s also mentioned in the allergy guidelines. Yes. It’s hard to say.* (Pair 21. Case labelled ‘barely similar’)	

## Discussion

### Summary of main findings

In this study we investigated the extent to which GP supervisors and GP trainees recognise each other’s EBM behaviour through observation, and we identified aspects that influence recognition. Our main finding is that the actor’s considerations are often not the same as what the observers recognises, and consequently EBM behaviour cannot be fully recognised by observation alone. Our analysis revealed several aspects connected to the observer, actor, consultation or pair that may enhance or hinder recognition of EBM behaviour through observation. These aspects are described in a model (Fig. 2). There were no specific differences between Dutch and Belgian pairs.

### Strengths and limitations

#### Strengths

To our knowledge, this study is unique in its approach to investigating informal workplace-based learning of EBM. The few previous studies on this topic looked at clinically integrated EBM training, which tries to adapt formal, explicit learning in such a way that it is applicable in the workplace [11, 28, 29]. In contrast, we studied observations in daily clinical practice, where it is assumed implicit learning takes place. A better understanding of these learning processes will allow us to tailor future educational interventions in GP practice.

Secondly, this study used VSI to collect data on thought processes during decision-making. This method encourages reflection, deepens the interview and can overcome recall bias [23–25, 30]. VSI is a very efficient way to discuss concrete considerations, thoughts and perceptions linked to a specific moment and thus minimise socially desirable answers that might be given if we had taken a more general or abstract approach to the topic. Thirdly, we conducted a rigorous analysis of the results, with rotating pairs of researchers from different professional backgrounds coding and labelling all cases. The vast number of cases (*n* = 85) enabled data saturation. The multicentre approach deepened the results and enhances transferability.

#### Limitations

Our results could be influenced by the participant sampling method. Given that the study bears a focus on EBM, GPs and trainees with a pronounced interest in EBM may have been more inclined to participate. Furthermore, the difficulties recruiting Dutch participants forced us to switch to convenience sampling. However, as the results show a wide range of attitudes to EBM among participants and the composition of the Dutch and Flemish group was comparable, we believe that self-selection bias and the convenience sampling in the Netherlands has had no significant impact on the results.

Selection bias may have played a role in the sampling of video-taped consultations, as participants may have chosen consultations which they expected would show off their ‘better’ EBM behaviour. To avoid this, we asked participants to record at least ten consultations, whereas we selected only two fragments for the interviews. This also minimised the risk of camera-related socially desirable behaviour, since previous research has shown that awareness of being filmed fades when the recording continues for a longer period of time [30, 31].

Secondly, the supervisor-trainee relationship might have prevented trainees from commenting on their supervisor’s behaviour in full honesty. We tried to prevent this by guaranteeing not to share the information given in the interviews with their supervisor, but we cannot be completely sure of the respondents’ perception of this.

### Implications of the findings in context of existing research

Our study showed that supervisors and trainees often have problems recognising EBM behaviour when they observe each other’s consultations. To our knowledge, this study is the first to use recognition of EBM considerations as a prerequisite for learning and thus sheds light on informal observational learning of EBM behaviour. Nevertheless, the aspects we identified can be linked to previous research.

#### Consultation-related aspects: recognition does not always require making EBM behaviour explicit during the consultation

Previous research on observational learning in the workplace reasoned that considerations should be made explicit to improve observational learning. When this is done, the observer will be able to *‘look into the actor’s head’*. [19] Finding few explicitly visible signs of EBM behaviour, Zwolsman (2013) suggested that making the decision-making process explicit would help observers recognise EBM behaviour and inform further learning [20]. Based on our findings, we question whether this is always the case. On the one hand, we observed that when a patient gives explicit input to the decision-making process, compelling the actor to make their considerations more explicit, the observer recognised this aspect of judicious decision-making more easily. This is in line with current thinking and findings on shared decision-making (SDM), which can be well observed and assessed by observing [32].

On the other hand, explicating during the consultation does not seem to be crucial for recognition of one another’s considerations: the observer sometimes missed the explicit cues or phrases mentioned by the actor during the consultation. However, implicit EBM behaviour was often recognised when the actor and observer had previous discussed the topic or shared the same knowledge. It can be concluded that although it is important to make considerations explicit to enhance SDM, it is not essential to improve the observer’s recognition of EBM behaviour [33]. Within-pair factors related to context, attitude or knowledge seem to have a greater influence on recognition.

#### Pair-related aspects: aligning attitudes and knowledge through discussion is crucial

Obtaining shared knowledge and having a shared attitude to EBM were important for both GP supervisor and trainee and led to recognition even without explicit mention of the actor’s argumentation during the consultation. Previous thinking on the role of background knowledge in observational learning by Csibra (2006) confirms the importance of having shared background knowledge. Csibra states that even basic skills, such as tool use, cannot be learned correctly through observation without adequate background knowledge. He explains, “*A behaviour can always be generated and explained by an infinite number of different mental state combinations, representing diverse goals and/or different types of background knowledge.”* [34] This means that observational learning of EBM behaviour cannot occur optimally without consensus on or insight into the knowledge that was used in the observed action. When straightforward topics (such as medical topics for which there are basic guidelines) play a crucial role in the consultation, individual achievement of this knowledge might be enough for adequate recognition of the considerations. However, since EBM behaviour often demands more complex skills and also relies partly on tacit knowledge, most of such shared knowledge probably needs to be constructed in social processes and through discourse [35]. Moreover, besides obtaining new, shared background knowledge, such dialogue gives a better understanding of the actor’s knowledge and attitude, leading to better recognition of implicit EBM behaviour during the consultation. Our results indicate that social and deliberative learning activities, such as dialogue, lead to better recognition of each other’s considerations, and are therefore essential for workplace-based learning of EBM behaviour.

#### Actor-related aspects: mindlines hamper correct observation

The ethnographers Gabbay and Le May stated that GPs and other clinicians often rely on internalised and collectively reinforced tacit guidelines during clinical reasoning and decision-making. Implicit guidelines, termed ‘mindlines’, are acquired in daily practice, in discussion with (expert) colleagues and reflection on own experiences. Exact elucidation of such tacit knowledge following decision-making is difficult [35, 36]. The concept of mindlines also arose in our study. Many participants found it difficult to recall their underlying considerations when asked to elaborate on them during the VSI. This occurred more often with experienced, older GP supervisors. Observers generally had problems recognising the EBM behaviour of clinicians who relied heavily on their mindlines, which led to no or erroneous recognition. It can be concluded that observational learning is less effective when GP supervisors and trainees overuse implicit, tacit knowledge such as mindlines. In this case, deliberative learning, such as follow-up discussions after observations, are needed even more to benefit from observing. Another advantage of deliberative dialogue is that it not only leads to a learning effect for the observer, who asks why the acting clinician showed certain EBM behaviour, but it can also foster the actor’s reflection on and explication of their own tacit knowledge and thus enhance evidence-based decision-making in both parties [37]. More research needs to be done on the best ways to train and educate both supervisor and trainee to be engaged in such dialogues in an optimal manner.

#### Observer-related aspects: observers do not observe objectively

As extensively investigated within social and cognitive psychology, ‘observing’ involves far more than just watching and imitating [16]. Research shows that observation is influenced by someone’s own views and opinions on the observed actions [14]. In epidemiological research, this is known as ‘observation bias’ and relates to the phenomenon that an observer sees what he or she expects or wants to see [38]. This is in line with our results: observers draw quick conclusions, based on their own cognitive framework, and also easily judge the acting clinician, based on their own opinions and preferences. This mechanism prevents them from objectively observing and recognising EBM behaviour and from learning outside of their own framework of knowledge. It is not surprising that GP supervisors and trainees observe like this: clinicians are trained to synthesise, deduce and filter information they obtain through observation during daily clinical practice. This is well explained by Wieringa**,** GP and researcher on EBM and mindlines: “*What we observe as clinicians is not reality itself but the reality exposed to our method of reducing or filtering the various potentially relevant streams of knowledge of which we are consciously or unconsciously aware and from those, constructing a picture of current reality.”* [35] This applies to the work of a clinician, but our results show that this also occurs when observing each other in a learning situation. Thus, simply recognising a decision and supposing the considerations that preceded this decision is not enough for adequate learning. When looking for adequate ways to address workplace-based EBM learning, the role of the observer should be carefully considered.

### Implications for workplace-based EBM learning

Our results show that it is incorrect to assume that EBM behaviour is learned in the GP apprenticeship simply by observing and other implicit learning processes. To best benefit from informal observation in workplace-based EBM learning, our results suggest focussing on improving observation skills as well as making room for explicit follow-up discussions between supervisor and trainee. Observation skills may be improved by making both GP supervisor and trainee aware of the fact that automatic judgements, based on their own cognitive framework, can hamper their observations. For supervisors it could be useful to draw a distinction between their ‘assessment’ role in direct observation and the informal, non-judgemental way of observing needed to recognise and jointly learn EBM behaviour.

Secondly, the role of taking the time to discuss and elaborate on evidence-based decision-making should be emphasised. It could be helpful to create explicit learning moments where both GP and trainee can learn from each other’s approaches through discussion and reflection. This would have a twofold goal: conversations on medical topics would not only enhance direct learning but also usefully support efficient informal observational learning in later phases, since it leads to shared background knowledge and attitude alignment. Lastly, such discussions can also be useful to unveil tacit knowledge derived from mindlines, which may benefit both supervisor and trainee. However, more research needs to be done on how best to structure such informal and formal discussion moments.

## Conclusion

GP trainees and supervisors do not fully recognise EBM behaviour through observing each other’s consultations. Factors influencing recognition are related to the observer, actor, consultation or pair. To improve recognition of EBM behaviour and thus benefit from informal observational learning at the workplace, trainees and supervisors need to be made aware of the automatic judgements that they make, based on their own cognitive framework. Creating explicit learning moments in which EBM decision-making can be discussed can be beneficial, since such moments can lead to shared background knowledge. Furthermore, such discussions can also be useful to unveil tacit knowledge derived from mindlines, which may benefit both supervisor and trainee. However, more research needs to be done on how best to structure such informal and formal discussion moments, taking into account existing theories on medical education and professional development.

## Supplementary information

**Additional file 1. **Interview guide. *Overview of the interview guide used during the video-stimulated elicitation interviews*

## Data Availability

The datasets used and/or analysed during the current study are available from the corresponding author on reasonable request.

## Abbreviations

EBM: Evidence-based medicine
EdG: Esther de Groot
GP: General practitioner / general practice
HS: High on similarity
HS: Hilde Suijker
KVR: Kaatje Van Roy
LS: Low on similarity
LW: Lisanne Welink
MLB: Marie-Louise Bartelink
NVMO: Nederlandse Vereniging voor Medisch Onderwijs / Dutch Society of Medical Education
PP: Peter Pype
RD: Roger Damoiseaux
VSI: Video-stimulated elicitation interview
