# Supplementary material for: Applying evidence-based medicine in general practice: a video-stimulated interview study on workplace-based observation
Source: BMC Fam Pract. 2020 Jan 8;21:5. doi: 10.1186/s12875-019-1073-x (PMC6950930; doi:10.1186/s12875-019-1073-x)
Supplement: Supplementary file 1 — Additional file 1. Interview guide. Overview of the interview guide used during the video-stimulated elicitation interviews [file 12875_2019_1073_MOESM1_ESM.docx]

**Additional file 1: Interview guide.**

*Overview of the interview guide used during the video-stimulated elicitation interviews*

**Main aims of the study**

| To explore how GP supervisors and trainees think they explicitly and implicitly apply and evaluate EBM during consultations |
| --- |
|  |
| To investigate how GP supervisors and trainees recognise and interpret each other’s use of EBM during observations of consultations |

| **Leading questions** | **Probing** |
| --- | --- |
| This study concerns the way your make decisions in practice. To make such decisions, you need medical knowledge But what do you do when your ready knowledge is falling short? Which choices or decisions do you make? | - How important are different sources of information for you? How do you weigh their importance? |
| You will now see a video fragment of one of your own consultations, which we selected because we think you made or discussed a decision. Please think back of this specific consultation when answering the next questions.   1. Can you guide me through your decision-making process during this consultation? 2. How do you decide which of these considerations you actually make explicit (to your patient)? | - Why did you make this decision? - Which information did you use that led to this decision? - What role did the patient’s preference play? - In what way did you incorporate evidence or guidelines? - How did your own clinical experience play a role during this consultation? - Would your decision-making have been differently with a different patient? Why? |
| (After watching a fragment of their supervisor/trainee)   1. Do you see moments of decision-making during this consultation, and if so, how did you identify such a moment? 2. What are your thoughts on why the other physician acted this way?   Imagine that you watched this fragment together with your supervisor/trainee during a learning conversation.   1. What would you like to discuss with each other, based on what you just saw? Why? | - How do you see that he/she is making a decision here? - Which elements played a role during this decision-making process, do you reckon? - Why were these elements important to the acting physician, do you think? |
